# Gingival fibromatosis: clinical, molecular and therapeutic issues

**DOI:** 10.1186/s13023-016-0395-1

**Published:** 2016-01-27

**Authors:** Katarzyna Gawron, Katarzyna Łazarz-Bartyzel, Jan Potempa, Maria Chomyszyn-Gajewska

**Affiliations:** 1 Microbiology Department, Faculty of Biochemistry, Biophysics and Biotechnology, Jagiellonian University, 30-387 Krakow, Poland; 2 Department of Periodontology and Oral Medicine, Jagiellonian University, Medical College, Institute of Dentistry, 30-387 Krakow, Poland; 3 Oral Health and Systemic Disease Research Group, School of Dentistry, University of Louisville, Louisville, KY USA

**Keywords:** Gingival fibromatosis, Etiology, Pathogenesis, Molecular mechanism, Management

## Abstract

Gingival fibromatosis is a rare and heterogeneous group of disorders that develop as slowly progressive, local or diffuse enlargements within marginal and attached gingiva or interdental papilla. In severe cases, the excess tissue may cover the crowns of the teeth, thus causing functional, esthetic, and periodontal problems, such as bone loss and bleeding, due to the presence of pseudopockets and plaque accumulation. It affects both genders equally. Hereditary, drug-induced, and idiopathic gingival overgrowth have been reported. Hereditary gingival fibromatosis can occur as an isolated condition or as part of a genetic syndrome. The pathologic manifestation of gingival fibromatosis comprises excessive accumulation of extracellular matrix proteins, of which collagen type I is the most prominent example. Mutation in the *Son-of-Sevenless-1* gene has been suggested as one possible etiological cause of isolated (non-syndromic) hereditary gingival fibromatosis, but mutations in other genes are also likely to be involved, given the heterogeneity of this condition. The most attractive concept of mechanism for drug-induced gingival overgrowth is epithelial-to-mesenchymal transition, a process in which interactions between gingival cells and the extracellular matrix are weakened as epithelial cells transdifferentiate into fibrogenic fibroblast-like cells. The diagnosis is mainly made on the basis of the patient’s history and clinical features, and on histopathological evaluation of affected gingiva. Early diagnosis is important, mostly to exclude oral malignancy. Differential diagnosis comprises all pathologies in the mouth with excessive gingival overgrowth. Hereditary gingival fibromatosis may present as an autosomal-dominant or less commonly autosomal-recessive mode of inheritance. If a systemic disease or syndrome is suspected, the patient is directed to a geneticist for additional clinical examination and specialized diagnostic tests. Treatments vary according to the type of overgrowth and the extent of disease progression, thus, scaling of teeth is sufficient in mild cases, while in severe cases surgical intervention is required. Prognosis is precarious and the risk of recurrence exists.

## Background

Gingival fibromatosis (GF) is a rare condition characterized by pathological, diffuse or local growth of gingiva. In severe cases functional, periodontal, esthetic and psychological problems may occur. The condition may be related to hereditary factors and occurs as a non-syndromic hereditary gingival fibromatosis (HGF) or as a part of a syndrome. It may also develop in susceptible individuals as a side effect of systemic medications, including the anti-seizure, immunosuppressant, or calcium channel blockers. In some cases the etiology of the enlargement remains unknown. Excessive accumulation of extracellular matrix (ECM) components seems to contribute to the pathologic manifestation of GF, however, the molecular mechanisms responsible for it remain undefined. The risk of recurrence and lack of non-invasive therapies for GF used in dental practice highlight the necessity of searching for novel alternate therapies for this condition. The aim of this article is to present an updated review of the clinical features, etiology, differential diagnosis, pathological mechanisms and management of GF.

## Review

### Disease name and synonyms

GF is also called gingivomatosis, gingival enlargement, gingival hyperplasia, gingival overgrowth (GO), elephantiasis gingivae, familial elephantiasis, gigantism of the gingiva, and congenital macrogingivae [1].

### Definition

GF is a condition characterized by the pathological growth of gingival tissue. It is also described as “gingival enlargement”, which comprises gingival hyperplasia and hypertrophy. Hyperplasia refers to an increased number of cells, and hypertrophy refers to an increase in the size of the individual cells. GF can present as HGF, which may appear as an isolated entity or as part of a genetic disease or syndrome, as drug-induced gingival overgrowth (DIGO, GO) or as idiopathic gingival fibromatosis (IGF).

### Epidemiology

#### GF associated with hereditary factors (non-syndromic)

HGF (GINGF, ORPHA 2024, MIM 135300) is a rare disease with unknown prevalence [2–4] (Table 1).

**Table 1:** Characteristics of HGF (non-syndromic) and its co-existence with rare genetic diseases and syndromes

Disease/syndrome	Synonyms	Prevalence	Inheritance	Chromosomal region/gene locus	Causing or candidate gene	Age of onset	Clinical hallmarks
Hereditary gingival fibromatosis, HGF, ORPHA 2024, MIM 135300 [4]	Autosomal dominant gingival fibromatosis, Autosomal dominant gingival hyperplasia, Hereditary gingival hyperplasia	unknown	GINGF			All ages	Slowly progressive hyperplasia of the maxillary and mandibular gingiva. Occurs with eruption of the permanent teeth, more rarely with the primary dentition or at birth [97–100, 102, 163, 164].
			Autosomal dominant	2p21-p22 [97]	SOS1* [100]		
			GINGF2				
			Autosomal dominant	5q13-q22	CAMK4* [98]		
			GINGF3				
			Autosomal dominant	2p23.3-p22.3 [102]	unknown		
			GINGF4				
			Autosomal dominant	11p15 [77]	unknown		
Gingival fibromatosis with craniofacial dysmorphism, ORPHA 2025, MIM 228560 [5]	-	<1/1 000 000	Autosomal recessive	unknown	unknown	Neonatal	Gingival fibromatosis, macrocephaly, bushy eyebrows with synophrys, hypertelorism, downslanting palpebral fissures, flattened nasal bridge, hypoplastic nares, cupid bow mouth, high arched palate [165–167].
Gingival fibromatosis with progressive deafness, ORPHA 2027, MIM 135550 [6]	Jones syndrome	<1/1 000 000	Autosomal dominant	unknown	unknown	Adults	Gingival fibromatosis, progressive sensorineural hearing loss [168, 169].
Gingival fibromatosis/hypertrichosis syndrome, HTC3, ORPHA 2026, MIM 135400 [13]	Congenital generalized hypertrichosis terminalis (CGHT), Hirsutism/congenital gingival hyperplasia syndrome, Hypertrichose avec ou sans hyperplasie gingivale, Hypertrichosis with or without gingival hyperplasia	unknown	Autosomal dominant, autosomal recessive	17q24.2-q24.3 [170]	ABCA5* [171]	Infancy, neonatal	Generalized gingival fibromatosis occurring at birth or during childhood, hirsutism, generalized hypertrichosis predominantly affecting the face, upper limbs and midback [170–175].
Ramon syndrome, ORPHA 3019, MIM 266270 [14]	Cherubism/gingival fibromatosis/intellectual disability	unknown	Autosomal recessive	unknown	unknown	Infancy	Gingival fibromatosis, cherubism (fibrous dysplasia of the maxilla and mandible), delayed tooth eruption, narrow palate, short stature, kyphosis, scoliosis, mental deficiency, hypertrichosis, epilepsy [176–178].
Zimmermann -Laband syndrome [9]	Gingival fibromatosis/hepatosplenomegaly/other anomalies, Laband syndrome	<1/1 000 000	Autosomal dominant	1q32.2, 3p, 8q: t(3;8)(p21.2;q24.3) [179] 3p14.3: t(3;17)(p14.3;q24.3) [180]	KCNH1* [181, 182]	Infancy, neonatal	Gingival fibromatosis, delayed tooth eruption, prominent mandible, high arched palate, broad nasal bridge, thick lips, thick eyebrows, synophrys, myopia, cataracts, cardiomyopathy, hepatomegaly, splenomegaly, scoliosis, hyperextensible fingers, hypoplastic distal phalanges, mental disability, seizures [179–184].
Infantile systemic hyalinosis (ISH), ORPHA 2176, MIM 236490 [7]	Murray-Puretic-Drescher syndrome, Puretic syndrome	<1/1 000 000	Autosomal recessive	4q21.21 [185, 186]	ANTXR2* [187, 188]	Childhood	Gingival fibromatosis, osteolysis, osteoporosis, osteopenia, recurring subcutaneous tumors, recurrent infections, joint contractures, diarrhea [185–191].
Juvenile hyaline fibromatosis (JHF), ORPHA 2028, MIM 228600 [8]	-					Antenatal, neonatal, infancy	
Oculodental syndrome, Rutherfurd type, ORPHA 2709, MIM 180900 [12]	Gingival hypertrophy/ Corneal dystrophy, corneal dystrophy with gum hypertrophy, Rutherfurd syndrome	<1/1 000 000	Autosomal dominant	unknown	unknown	Infancy, neoneatal	Gingival fibromatosis, delayed primary teeth eruption, failure of secondary teeth eruption, corneal dystrophy, aggressive behaviour [192–194].
Amelogenesis imperfecta/nephrocalcinosis syndrome, ORPHA 1031, MIM 204690** [10]	Enamel - renal syndrome (ERS), Enamel - renal - gingival syndrome	<1/1 000 000	Autosomal recessive	17q24.2 [154, 195, 196].	FAM20A* [154, 195–197]	Orodental phenotype – childhood; renal phenotype - adults	Orodental phenotype: gingival fibromatosis, delayed tooth eruption, thin hypoplastic or absent enamel, microdontia and spaced teeth, intra-pulpal calcifications, root dilacerations of impacted teeth [154, 195, 196, 198]. Renal phenotype: bilateral medullary nephrocalcinosis, focal clusters of sclerosed glomeruli, marked periglomerular fibrosis with lymphocytic and plasma cell infiltration of the renal interstitium [199–202].
Amelogenesis imperfecta/ gingival fibromatosis syndrome (AIGFS), ORPHA 171836, MIM 614253** [11]							

*MIM*, Mendelian Inheritance in Man; *SOS-1, Son-of-Sevenless-1; *CAMK, calcium/calmodulin-dependent protein kinase IV; *ABCA5, ATP-binding cassette, subfamily A, member 5; *KCNH1, potassium channel, voltage-gated, subfamily H, member-1; *ANTXR2, anthrax toxin receptor 2; *FAM20A, family with sequence similarity 20, member A.

**Considering the significant overlap in the oral phenotype between cases with amelogenesis imperfecta (AI) with hamartomas and unerupted teeth, amelogenesis imperfecta/gingival fibromatosis syndrome (AIGFS), and enamel-renal syndrome (ERS) in the published literature as well as the pathognomonic character of the oral phenotype in the absence of other developmental health problems, the two OMIM entries for ERS and AIGFS (ERS:MIM#204690 and AIGFS:MIM#614253) have been combined [198]

#### GF associated with genetic diseases and syndromes

GF may also co-exist with rare genetic syndromes and diseases, *i.e.* GF with craniofacial dysmorphism (ORPHA 2025, MIM 228560) [5], GF with progressive deafness (ORPHA 2027, MIM 135550) [6], infantile systemic hyalinosis (ISH, ORPHA 2176, MIM 236490) [7], juvenile hyaline fibromatosis (JHF, Murray-Puretic-Drescher syndrome, ORPHA 2028, MIM 228600) [8], Zimmermann-Laband syndrome (ZLS, ORPHA 3473, MIM 135500) [9], amelogenesis imperfecta/nephrocalcinosis syndrome (ORPHA 1031, MIM 204690) [10], amelogenesis imperfecta/GF syndrome (AIGFS, ORPHA 171836, MIM 614253) [11], and oculodental syndrome (Rutherfurd syndrome, ORPHA 2709, MIM 180900) [12], which occur with a prevalence of one or less per million population. The frequency of other genetic entities that present with a periodontal phenotype, * i.e.* GF/hypertrichosis syndrome (ORPHA 2026, MIM 135400) [13] and Ramon syndrome (ORPHA 3019, MIM 266270) [14], is unknown (Table 1).

#### DIGO

The reported incidence of GF induced by the anti-epileptic drug, phenytoin could be as high as 70 % [15]. It also occurs in 15–83 % of patients treated with nifedipine [16, 17], 21 % of patients taking diltiazem [18], and approximately 4 % of patients cured with verapamil [19], while the prevalence rate of gingival enlargement in patients treated with cyclosporine A (CsA) is estimated at between 8 and 70 % [20–22].

#### IGF

IGF is known to affect 1 in 750,000 individuals, and can occur in both genders and in either of the jaws [1, 23, 24].

### Clinical description

**Fig. 1:**
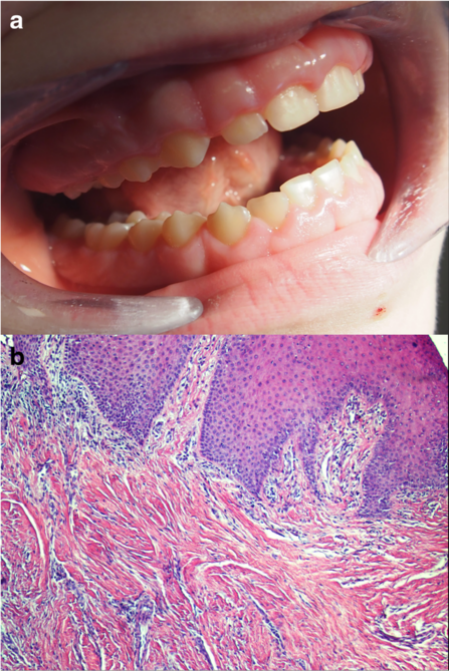
Diffuse, hereditary gingival fibromatosis in a 13-year-old female reported to the Department of Periodontology and Oral Medicine, Jagiellonian University, Collegium Medicum, Krakow, Poland. **a** Clinical appearance of the lesion before surgery. **b** Epithelial acanthosis, dense connective tissue consisting of numerous collagen fiber bundles, a moderate amount of fibroblasts, and scanty blood vessels in tissue sections stained by hematoxylin and eosin, original magnific. 100×. Histological staining was performed at the Microbiology Department, Jagiellonian University, Krakow, Poland

**Fig. 2:**
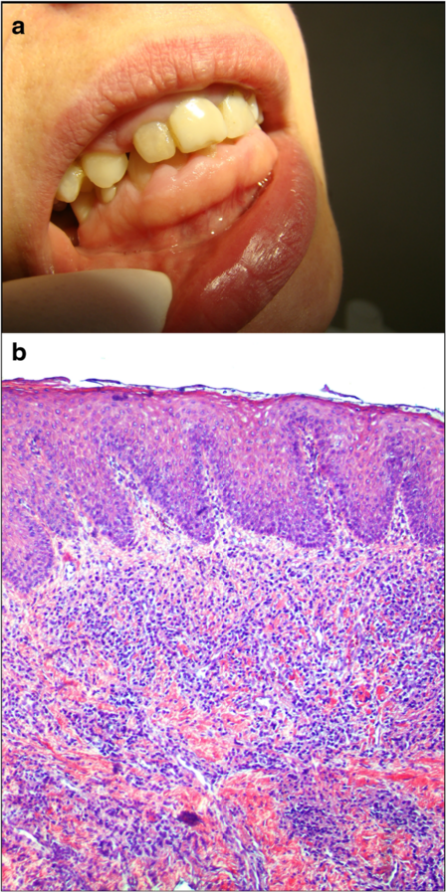
Non-syndromic, diffuse, hereditary gingival fibromatosis in a 32-year-old female treated at the Department of Periodontology and Oral Medicine, Jagiellonian University, Collegium Medicum, Krakow, Poland. **a** Recurrence at 1 year post-surgery is visible in the anterior region of the mandibula. **b** Hematoxylin and eosin staining shows epithelial acanthosis and atypically abundant inflammatory infiltrates distributed in the subepithelial and connective tissue, original magnific. 100×. Histological staining was performed at Microbiology Department, Jagiellonian University, Krakow, Poland [41]

**Fig. 3:**
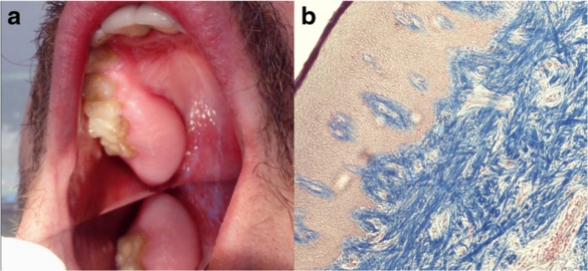
Idiopathic gingival fibromatosis in the posterior maxillary region of the gingiva of a 35-year-old patient treated at the Department of Periodontology and Oral Medicine, Jagiellonian University, Collegium Medicum, Krakow, Poland. **a** Clinical image of the lesion before surgery. **b** Numerous bundles of collagen fibrils oriented in antithetic directions in the dense, thick connective tissue, visualized by Heidenhain’s trichrome staining, original magnific. 100×. Histological study was conducted at Microbiology Department, Jagiellonian University, Krakow, Poland [37]

The most common form of GF occurs as a benign, slowly progressive and non-hemorrhagic enlargement of the gingiva. It affects the masticatory mucosa (the marginal and attached gingiva and the interdental papilla), but it does not spread beyond the muco-gingival junction. GF can be generalized, idiopathic or hereditary (non-syndromic) (Figs. 1a and 2a) or associated with different genetic diseases. Clinically, the onset coincides with the eruption of primary or permanent dentition, and rarely presents at birth [25]. GF may also occur as a local, nodular-like lesion. The excess gingival tissue can cover part of or the entire crown, and can result in diastemas, teeth displacement, or retention of primary or impacted teeth, and may also cause masticatory, phonetic, psychological, and esthetic problems [26]. An example of a local, nodular-like lesion in the posterior maxillary region of the gingiva in a 35-year-old patient diagnosed as an IGF is depicted in Fig. 3a. Clinical features of isolated HGF, and genetic syndromes and diseases co-existing with HGF are presented in Table 1. DIGO usually occurs as a generalized (diffuse) enlargement usually visible within several months after the onset of systemic therapy. This is in contrast to HGF, which is characterized by a slow, progressive growth of the gingival tissue. GO may vary from mild to severe, depending on the dose of the drug. Combination therapy, such as with amlodipine, a calcium channel blocker used in the management of hypertension and angina, and cholesterol-lowering drugs, can result in more severe GO than single-agent therapy [17]. Although the clinical appearance of lesions resulting from different medications is similar, the histopathology of DIGO lesions varies depending on the inducing drug [27]. Hyperplastic gingiva usually presents a normal coloration or can be erythematous. Periodontal problems, such as bleeding and bone loss, might occur due to the excess of gingival tissue, presence of pseudopockets and plaque accumulation [17, 28–30].

### Histopathological description

The typical histopathology of the lesion involves hyperplasia of the epithelium with elongated rete ridges extending into the underlying connective tissue [31–34] (Figs. 1b and 2b). The connective tissue consists of excess collagen, but has relatively few fibroblasts and blood vessels [35, 36] (Fig. 1b). Enlarged fibroblasts appear to alternate with thin and thick collagen fibrils. Elastic and oxytalan fibers are also present in GF lesions. Unlike in normal gingiva, coarse and fine dense collagen fiber bundles are oriented in all directions [32, 33, 37] (Fig. 3b). Small osseous calcifications and abundant neurovascular bundles may also be present. The excess of gingival tissue may provide new niches for the growth of microorganisms, plaque accumulation and pseudopockets formation resulting in inflammatory infiltration of the gingival connective tissue [17, 38–41] (Fig. 2b).

### Diseases associated with “gingival enlargement”

Gingival enlargement covers broad etiological entities classified into five general groups:

1. Enlargement associated with non-genetic diseases
  GF can be directly or indirectly linked to poor nutrition (vitamin C deficiency) [42], systemic hormonal stimulation (pregnancy or puberty) [43], blood dyscrasias (leukemia) [44–47], Wegener’s granulomatosis [48], orofacial granulomatosis [49], pyogenic granuloma [50] and sarcoidosis [51]. It may also be associated with pseudotumors [52, 53], benign neoplasms, * e.g*. giant cell fibroma [54], gingival and oral myofibroma [55–57], papilloma [58], giant cell granuloma [59], and malignant neoplasms, *e.g.* oral squamous cell carcinoma [60, 61], salivary gland tumors [62, 63], melanoma [64–67], adenoma and mucoepidermoid carcinoma [68].
2. Enlargement associated with inflammatory diseases of the oral cavity
  Gingival enlargement may develop during the course of inflammatory diseases of the oral cavity, for example localized and generalized aggressive periodontitis (AP) and primary gingival tuberculosis [69–73]. Plaque accumulation and bacterial infection resulting from poor oral hygiene are significant predisposing factors [41, 74]. Other examples are inflammatory pseudotumors [53] and inflammatory fibrous hyperplasia due to local irritants [75].
3. Enlargement associated with hereditary factors and co-existing with genetic diseases and syndromes [4, 9–14, 25, 35, 41]
4. Drug-induced gingival enlargement [15–18, 20, 39]
5. Gingival enlargement of unknown etiology [1, 37, 40, 76]

### Etiology

#### GF associated with hereditary factors (non-syndromic and associated with genetic diseases and syndromes)

HGF (ORPHA 2024, MIM 135300) is a rare and slowly progressive condition characterized by etiological heterogeneity [4, 23, 29, 41, 77]. Additionally, it may co-exist with several genetic diseases or syndromes (Table 1). Moreover, it may occur sporadically in several other syndromes and diseases. An example is Cowden syndrome (multiple hamartoma syndrome, CWS1-6, ORPHA201, MIM 158350) [78], a rare autosomal dominant disorder characterized by multiple hamartomas and a high risk of development of malignancy. It is now believed that 25 % of CWS cases (CWS1) are caused by germline mutations in the phosphatase and tensin homolog (*PTEN*) gene (10q23), which encodes PTEN, a dual-specificity phosphatase. Patients with CWS and CWS-like phenotypes without PTEN involvement have been found to have germline promoter methylation of *KLLN* (10q23; CWS4) (30 % of cases); germline variations in *SDHB* (1p36; CWS2), *SDHC* or *SDHD* (11q23; CWS3) (10 % of cases); or germline mutations in *AKT1* (14q32; CWS6) or *PIK3CA* (3q26; CWS5) (10 % of cases) [79–82]. The most predominant features of the syndrome are small papular cutaneous lesions, papillomatous outgrowth, and fibromas of the oral mucosa and tongue [83, 84]. The co-existence of gingival hyperplasia with Cowden syndrome was reported by two groups [85, 86]. Another example is Bardet-Biedl syndrome (BBS1-19, ORPHA 110, MIM 209900) [87], a rare, heterogeneous, oligogenic or autosomal recessive condition with a prevalence in Europe estimated at between 1/125,000 and 1/175,000. Clinical features include obesity, pigmentary retinopathy, post-axial polydactyly, polycystic kidneys, hypogenitalism, and learning disabilities, many of which appear several years after disease onset. The clinical expression is variable, but most patients exhibit the majority of these clinical features during the disease course [88–91]. Dental anomalies, regarded as secondary manifestations, include hypodontia, microdontia, short roots, and a deep palate. The first case of BBS with generalized GO was reported by Drugowick et al. [92]. An example of a disease where genetic and infective factors are involved is AP. It is a rare, severe, and rapidly progressive form of periodontitis that develops as a result of complex interactions between specific host genes and the local microenvironment of the oral cavity. Factors which increase the risk of AP development include: familial aggregation, single nucleotide polymorphisms, functional defects in neutrophils, antibodies to specific bacteria, herpes virus infection, stress and smoking [69]. Though GF and AP usually occur as separate entities, several reports describe the co-existence of these diseases. The first report was published by Mahajan et al. [70], followed by reports by Jadwat et al. [71], Chaturvedi [93], Sandhu et al. [94], and Vishnoi and Phadnaik [72]. Recently, Ramachandra et al. [95] reported a case of gingival enlargement associated with generalized AP and the presence of mesiodens. The common features of GF and localized AP are their onset around puberty, female predilection, hereditary background, and progression in the presence of minimal local factors, although secondary involvement can aggravate the pre-existing condition [96].

HGF may be transmitted as a Mendelian trait in an autosomal dominant or, less commonly, an autosomal recessive fashion [29]. Linkage analysis has provided clues about the etiology of the disease, and showed several chromosomal regions that may contain mutations responsible for HGF. Loci for isolated, non-syndromic autosomal dominant forms of HGF have been localized to chromosome 2p21-p22 in a Brazilian family [97], and to chromosome 5q13-q22 [98] and 11p15 in a number of Chinese families [99]. The candidate loci are *GINGF*, *GINGF2* and *GINGF4*, respectively.

After sequencing 16 genes in the candidate interval (2p21-p22), a single base insertion in the Son-of-Sevenless-1 (*SOS-1*) gene (MIM 182530) underlying *GINGF* locus, was detected in a Brazilian family [100], but not in three Chinese families [101]. Similar to the observations of Ma et al., our recent study did not confirm the presence of a single nucleotide insertion in the *SOS-1* gene in two Polish families diagnosed with non-syndromic HGF (unpublished data). Interestingly, in four other Chinese families, non-syndromic HGF was linked to a mutation in the 2p21 locus (*GINGF*), as previously reported by Hart et al. [97]. A mutation in the *SOS-1* gene has been suggested as one possible etiological factor/causing gene for isolated, non-syndromic HGF, but considering the genetic heterogeneity of HGF, mutations in other genes are also likely to be involved.

By haplotype construction and analysis in a five-generation Chinese family segregating autosomal dominant HGF, Ye et al. [102] identified a novel locus, which they designated *GINGF3*, in an 11.4 cM interval between markers D2S2221 and D2S1788 on chromosome 2p23.3-p22.3. Authors noted that this locus is distal to and does not overlap with the previously described *GINGF* locus on 2p22-p21 [102].

#### DIGO

Drug-induced GF occurs in susceptible individuals as a side effect of systemic medications, including the anti-epileptic drug phenytoin, the immunosuppressant CsA, and calcium channel blockers, namely, dihydropyridines, particularly nifedipine, diltiazem, and verapamil, which are widely used to control hypertension [103, 104]. Currently, more than 20 drugs are associated with gingival enlargement [17, 105]. Although the drugs that can cause GO have distinct mechanisms of action and act on different primary target tissues, they all seem to have a similar adverse effect on the gingival connective tissue [22, 104, 106]. In addition to being disfiguring, GO may also lead to poor oral hygiene and decreased oral function in individuals already suffering from conditions such as epilepsy, cardiovascular diseases, or immunosuppression [107]. The pathogenesis of DIGO is dependent on several factors, such as: age, genetic predisposition, pharmacokinetic variables, alterations in gingival connective tissue homeostasis, pre-existing dental plaque and gingival inflammation, and interaction of drugs and growth factors [106, 108, 109].

### Pathogenesis and Pathophysiology

The pathologic manifestation of GF is excessive accumulation of ECM proteins, including collagen type I [110–114]. Thus far, however, the molecular and biochemical mechanisms that trigger this pathological process are not completely understood.

During collagen biosynthesis, nascent single procollagen polypeptides undergo post-translational modification in the endoplasmic reticulum (ER), form triple-helical chains, and are secreted into the extracellular space. This process involves heat shock protein 47 (HSP47), a 47 kDa glycoprotein localized in the ER. It binds to the nascent type I procollagen peptides to prevent premature folding and aggregation of procollagen chains, and participates in the translocation and secretion of procollagen I into the extracellular space [115]. Type I collagen and HSP47 mRNA and protein levels are increased significantly in fibroblast cultures derived from patients with HGF [116]. Moreover, transforming growth factor (TGF)-β1 and interleukin (IL)-6 induce the expression of type I collagen and HSP47 and to downregulate matrix metalloproteinase (MMP)-1 and MMP-2 in fibroblast cultures from HGF patients. The effect of TGF-β1 and IL-6 on the synthesis of other ECM proteins was also reported in DIGO [112, 117–120]. By contrast, interferon-γ (IFN-γ) reduced collagen I and HSP47 expression, and it slightly affected MMP-1 and MMP-2 expression [116]. This observation suggests that HSP47 may be a crucial molecule in the post-translational processing of the overproduced type I procollagen chains, while enhanced TGF-β1 and IL-6 production in patients with GF may favor the accumulation of collagen fibrils in the gingiva.

Prolyl 4 hydroxylases (P4Hs) are equally essential enzymes in the biosynthesis and folding of newly-synthesized collagen polypeptide chains into triple-helical molecules [121]. P4Hs are α_2_β_2_ tetramers consisting of one of three isoforms of subunit α [α(I), α(II), or α(III)] that have similar catalytic activity [122]. The expression of the α subunit of P4Hs limits the rate of active P4H formation, P4H activity, and collagen synthesis [123]. Increased levels of P4H activity are reported in a number of fibrotic conditions such as keloids and hepatic fibrosis [124, 125]. Some studies report increased expression and activity of prolyl hydroxylases in IGF, DIGO, and HGF [126, 127]. Notably, Meng et al. [127] identified isoform I of the α subunit of P4Hα as the isoform associated with GO, while the type II and III forms of P4H were not affected. These findings suggest that P4Hα (I) might be involved in the pathogenesis of HGF and confirm that HGF fibroblasts are deregulated at the level of post-translational protein modification.

Alterations in the expression of MMPs, key enzymes regulating the composition of the ECM, have been implicated in the pathogenesis of GF. Several studies show a significant decrease in the expression and activity of MMP-1 and MMP-2 in fibroblasts from HGF patients in comparison with controls [116, 128]. MMP-1 is a collagenase that degrades interstitial collagen, while MMP-2 acts predominantly on type IV collagen, but it has also been shown to degrade type I collagen in its native form [129]. Similarly, the inhibition of MMP-1, MMP-2, and MMP-3 has been reported in CsA-induced GO, a condition also associated with enhanced TGF-β1 production [114, 130, 131]. The catalytic activity of the MMPs is regulated at the transcriptional level as well as by tissue matrix metalloproteinase inhibitors (TIMPs). Interestingly, addition of anti-TGF-β1 antibodies in the study by Coletta et al. [128] resulted in a slight increase in MMP-1 and a decrease in MMP-2 expression, whereas TIMP-1 and TIMP-2 expression were unaffected. These results confirm previous observations that enhanced TGF-β1 production may lead to the accumulation of ECM by altering the proteolytic activities of fibroblasts.

Systemic therapy with CsA, phenytoin, and nifedipine modulates cytokine levels and indirectly affects gingival connective tissue metabolism. For example, Hong and Trackman [132] observed that the mRNA expression of lysyl oxidase and collagen type I by human gingival fibroblasts decreased to 53 % and to less than 10 % of control levels, respectively, after 48 h of treatment with 1 nM basic fibroblast growth factor (bFGF), while lysyl oxidase enzymatic activity was downregulated by 10–20 %. By contrast, interleukin - 1 (IL-1), IL-6, and platelet-derived growth factor-BB (PDGF-BB) did not significantly regulate the enzymatic activity of lysyl oxidase or the mRNA levels of lysyl oxidase, collagen type I or elastin [132]. The opposite effect was found by stimulation of gingival fibroblasts with TGF-β1. In brief, TGF-β1 upregulated lysyl oxidase and collagen type I, but not elastin, in a dose- and time-dependent manner. The maximal effect on lysyl oxidase activity and mRNA expression, as well as the mRNA expression of collagen I occurred after 48 h of treatment of gingival fibroblastic cells with 400 pM TGF-β1.

It has also been shown that mRNA and protein synthesis of connective tissue growth factor (CTGF or CCN2) are significantly induced by TGF-β1 in human gingival fibroblasts [133]. CTGF/CCN2 is a member of the CCN family, members of which contain conserved cysteine-rich domains and have a variety of biological activities [134]. CTGF promotes the proliferation of various cell types [135, 136] and is highly expressed in a wide variety of fibrotic lesions, including skin and kidney fibrosis, and atherosclerosis [137–139]. Uzel et al. [27] assessed the expression and localization of CTGF in GO induced by three different systemic medications. CTGF expression was significantly higher in phenytoin-induced GO than in CsA- or nifedipine-induced GO. Similar observations are reported by Hong et al. [133]. Considering that phenytoin-induced lesions are more fibrotic than nifedipine- and CsA-induced lesions, it seems that CTGF levels correlate positively with fibrosis and have a role in promoting and maintaining fibrosis. Development of fibrotic events can involve the process known as epithelial/mesenchymal transition (EMT). It occurs physiologically during organ and tissue development. In this process, partial destruction of the basement membrane can lead to inappropriate diffusion of factors between the connective tissue and the epithelial layers of gingival tissues. These factors can stimulate epithelial cells to lose cell-cell contacts, decrease E-cadherin expression, and increase cell motility, promoting their invasion into the underlying connective tissue stroma, where they differentiate further into cells that are indistinguishable from fibroblasts and myofibroblasts. These fibroblastic cells, in turn, produce connective tissue proteins that contribute to fibrosis [140–142].

### Diagnosis

A diagnosis of GF is made mainly on the basis of clinical and periodontal examination, a medical and family history and laboratory tests. Clinical examination, medical history and laboratory tests determine initially whether the condition is inherited or acquired, the presence of other diseases, the prior therapies used, and the involvement of primary dentition. Histopathological analysis characterizes the typical features of fibromatotic gingiva, such as the rate of epithelial acanthosis, connective tissue density and cellular content, the extent of fibrosis or inflammatory infiltrates. Periodontal examination, including X-ray and histopathology serve mainly to assess the type (local, diffuse, fibrous or inflammatory) and the severity of gingival involvement, including bone erosion, and allow the physician to choose the optimal treatment option [143–146].

### Differential diagnosis

If a genetic background is found, it is important to verify whether the lesion is an isolated entity or occurs as part of a multisystem pathology. The list of the syndromes associated with periodontal involvement is presented in Table 1. Particularly important are neoplasms, though benign tumors and pseudotumors can given similar patterns of clinical involvement [52]. Amongst benign tumors of the gingiva, giant cell fibroma, irritation fibroma, neurofibroma, angiofibroma (tuberous sclerosis or Bourneville-Pringle disease), inflammatory myofibroblastoma and epulis fissuratum should be considered. Epulis fissuratum is a specific condition usually found in patients with removable full dentures. Inflammatory myofibroblastoma ranges from completely benign to malignant tumors with a fatal outcome [53–57, 147, 148]. Malignant neoplasms which have to be considered are oral squamous cell carcinoma [60, 61], salivary gland adenocarcinoma [62, 63], melanoma 64–67], adenoma and mucoepidermoid carcinoma [68]. Regarding DIGO, it may occur not only for calcium channel blockers, CsA and phenytoin but also with other immunosuppressants or anticonvulsants, antibiotics, and oral contraceptives [149]. Differential diagnosis of GF with granulomatous lesions includes systemic diseases such as sarcoidosis, Crohn’s disease, and tuberculosis, as well as lesions localized to the orofacial region like orofacial granulomatosis [48, 49, 51, 150]. Although leukemia is a malignant disease of the blood, where the uncontrolled proliferation of immature blood cells takes place, leukemic infiltration of oral tissues may occur, resulting in a pale mucosa, poor wound healing, and bleeding, which in some cases can resemble GF [45, 46]. Lymphomas rarely manifest initially in the oral cavity, but misdiagnosis is highly probable because maxillary and mandibular swelling can mimic fibrotic lesion [47, 151]. All these conditions require thorough analysis of medical history, laboratory tests, and histopathology as a delay in the diagnosis, particularly in the case of malignancy may worsen the prognosis.

### Genetic counseling

HGF may present as an autosomal-dominant or less commonly autosomal-recessive mode of inheritance, as an isolated disorder or as part of a syndrome [4, 9, 10, 13, 152]. Autosomal dominant forms are usually isolated (non-syndromic) and have been genetically linked to several loci, *i.e.* GINGF, GINGF2, GINGF3, GINGF4 [97–100, 102] (Table 1). If clinical/periodontal examination, family history and laboratory tests initially indicate a genetic background, other family members are called to the clinic to confirm the presence of HGF, draw up a pedigree diagram, and determine whether it constitutes an isolated entity or co-exists with another disease or syndrome. If a systemic disease or syndrome is suspected, the patient is directed to a geneticist for additional clinical examination and specialized diagnostic tests. In such cases psychological support is needed to assess a risk of occurrence in future pregnancies, the option of early prenatal diagnosis and optimal treatment.

### Management including treatment

The patient’s medical history (*e.g.* patient’s age and the presence of other diseases) and the findings of the clinical examination (*e.g.* the type and severity of overgrowth) influence the patient’s management. While some surgical approaches, such as the use of laser excision, reportedly reduce the recurrence, re-growth of the excised gingival tissue due to the continuous use of the drug presents a significant challenge [153]. The usual treatment of GF includes external bevel gingivectomy using a scalpel, unless this is complicated by bony defects, in which case, a flap surgery is carried out. The surgery is followed by 0.12 % chlorhexidine oral rinses twice a day for 2 weeks. Removal of the hypertrophic tissue can be also done by electrosurgery or by laser, reducing the risk of bleeding and pain. This type of procedure decreases significantly the quantity of local anesthetic used, leads to better visibility, which reduces the chairside time, and results in better patient acceptance [74, 154, 155]. Non-surgical treatment includes scaling and root planing, oral hygiene instructions and administration of antibiotics, usually amoxicillin and metronidazole, along with anti-inflammatory (ibuprofen) and analgesic (paracetamol) drugs and the use of chlorhexidine mouth rinses. At the end of the fourth week, internal bevel gingivectomy along with open flap debridement is carried out. This procedure eliminates the pocket, reduces the bulk of the tissue and makes plaque control much easier. Management of patients diagnosed with GF and AP includes non-surgical treatments, surgery with regenerative or resective therapy and anti-microbial treatment. Regenerative techniques include the use of bone grafts, barrier membranes, wound healing agents and enamel matrix protein. Local drug delivery, full mouth disinfection and host immune response modulation are other modes of treatment [38, 72, 76, 95, 156–158].

Because of difficulties in the treatment of DIGO, the status of oral health prior to and during drug administration, in combination with drug serum levels and the duration of therapy, are key factors in the management of the condition. Discontinuing or dramatically lowering the dose of the drug often results in resolution of clinical signs and lesions. However, this is not always medically feasible, particularly in whole-organ transplant recipients.

Evidence suggests that 34 % of cases demonstrate recurrence during the 18 months following periodontal surgery regardless of the drug [156]. Although GO lesions are not directly life-threatening and may be tolerated by some patients without treatment, the quality of life is clearly compromised among affected individuals. Therefore, to stabilize the long-term outcomes and alleviate suffering for those who are adversely affected, non-surgical therapies to treat GO are of importance. Although progress in the clinical management of human GO has been made in relatively affluent societies, approaches of drug substitutions and careful dose adjustments are not universally practiced by physicians worldwide, and this contributes to the global public health impact of GO [157, 159].

### Prognosis

Complications related to GF include difficulties with mastication, speech problems, displacement of teeth, esthetic effects, and psychological difficulties for the patient; therefore, appropriate treatment and postoperative management are crucial. Gingival enlargement as a form of periodontal tissue reaction may impose a challenge to periodontists as well. Routine treatment of minimal and local enlargements relies on keeping appropriate oral hygiene and/or root scaling, while cases of advanced, diffuse gingival enlargement require surgical intervention. Recurrence can occur several months to several years after surgery [36, 41, 160–162].

## Conclusions

GF is a rare and slowly progressive condition that is also characterized by etiological heterogeneity. Additionally, this condition constitutes a typical symptom of several genetic syndromes, and it may occur sporadically in several other syndromes and diseases, as reported recently. By contrast, DIGO may occur as soon as several months from the onset of systemic therapy in susceptible individuals treated with certain anti-seizure drugs, immunosuppressants or calcium channel blockers. Diagnosis is made based on medical history, clinical examination, blood tests and histopathological evaluation of affected gingival tissue. Differential diagnosis includes consideration of all pathologies in the mouth that involve excessive accumulation of gingival tissue, including syndromic HGF. In general, the histological features of GF are similar, but phenytoin-induced GO is reported to be most fibrotic and to express higher levels of CTGF than nifedipine- and CsA-induced GO. Excessive accumulation of ECM components, particularly collagen type I, seems to contribute to the pathologic manifestation of all etiological types of GF; however, the molecular mechanisms responsible for it remain undefined. Further studies concerning interactions among medications, the innate and acquired immune response, cytokines and growth factors, and gingival epithelial and connective tissue cells are essential for a better understanding of the detailed molecular and mechanistic pathways controlling the unique metabolism of gingival connective tissue. This would improve disease management and allow the implementation of less invasive therapeutic methods than surgery into routine dental practice.

## Abbreviations

*ABCA5*: ATP-binding cassette, subfamily A, member 5
AIGFS: amelogenesis imperfecta/gingival fibromatosis syndrome
*AKT1*: v-akt murine thymoma viral oncogene homolog 1
*ANTXR2*: anthrax toxin receptor 2
AP: aggressive periodontitis
BBS: Bardet-Biedl syndrome
bFGF: basic fibroblast growth factor
*CAMK*: calcium/calmodulin-dependent protein kinase IV
CGHT: congenital generalized hypertrichosis terminalis
CsA: cyclosporine A
CTGF/CCN2: connective tissue growth factor
CWS: Cowden syndrome
DIGO: drug-induced gingival overgrowth
ECM: extracellular matrix
EMT: epithelial/mesenchymal transition
ER: endoplasmic reticulum
ERS: enamel-renal syndrome
*FAM20A*: family with sequence similarity 20, member A
GF: gingival fibromatosis
GO: gingival overgrowth
HGF: hereditary gingival fibromatosis
HSP 47: heat shock protein 47
HTC3: gingival fibromatosis/hypertrichosis syndrome
IFN γ: Interferon γ
IGF: idiopathic gingival fibromatosis
IHC: immunohistochemistry
IL-1: Interleukin-1
IL-6: Interleukin-6
ISH: infantile systemic hyalinosis
JHF: juvenile hyaline fibromatosis
*KCNH1*: potassium channel, voltage-gated, subfamily H, member-1
*KLLN*: killin, p53-regulated DNA replication inhibitor
MIM: mendelian inheritance in man
MMP: matrix metalloproteinase
P4H: prolyl 4 hydroxylase
PCNA: proliferating cell nuclear antigen
PDGF-BB: platelet-derived growth factor-BB
*PIK3CA*: phosphatidylinositol-4,5-bisphosphate 3-kinase, catalytic subunit alpha
*PTEN*: phosphatase and tensin homolog
*SDHB*: succinate dehydrogenase complex, subunit B, iron sulfur (Ip)
*SDHC*: succinate dehydrogenase complex, subunit C, integral membrane protein
*SDHD*: succinate dehydrogenase complex, subunit D, integral membrane protein
*SOS-1*: Son-of-Sevenless-1
TGF-β_1_: transforming growth factor-β_1_
TIMP: tissue matrix metalloproteinase inhibitor
ZLS: Zimmermann-Laband syndrome
